# Supplementary material for: Inequalities in the prevalence of cardiovascular disease risk factors in Brazilian slum populations: A cross-sectional study
Source: PLOS Glob Public Health. 2022 Sep 8;2(9):e0000990. doi: 10.1371/journal.pgph.0000990 (PMC10022010; doi:10.1371/journal.pgph.0000990)
Supplement: S1 Fig — (DOCX) [file pgph.0000990.s001.docx]

**S1 Fig. Figures on post-regression predicted prevalence of risk factors by region and area of residence (urban slum, urban non-slum, rural).**

| 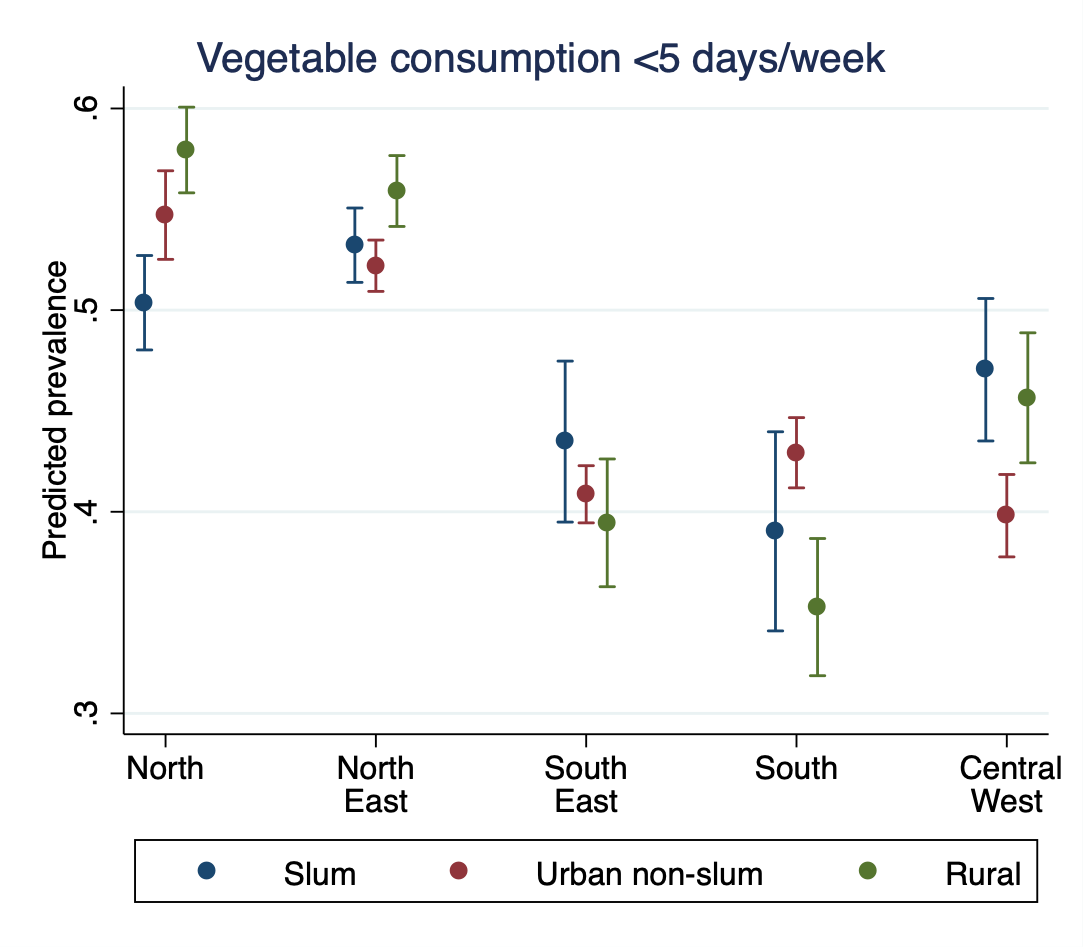 | 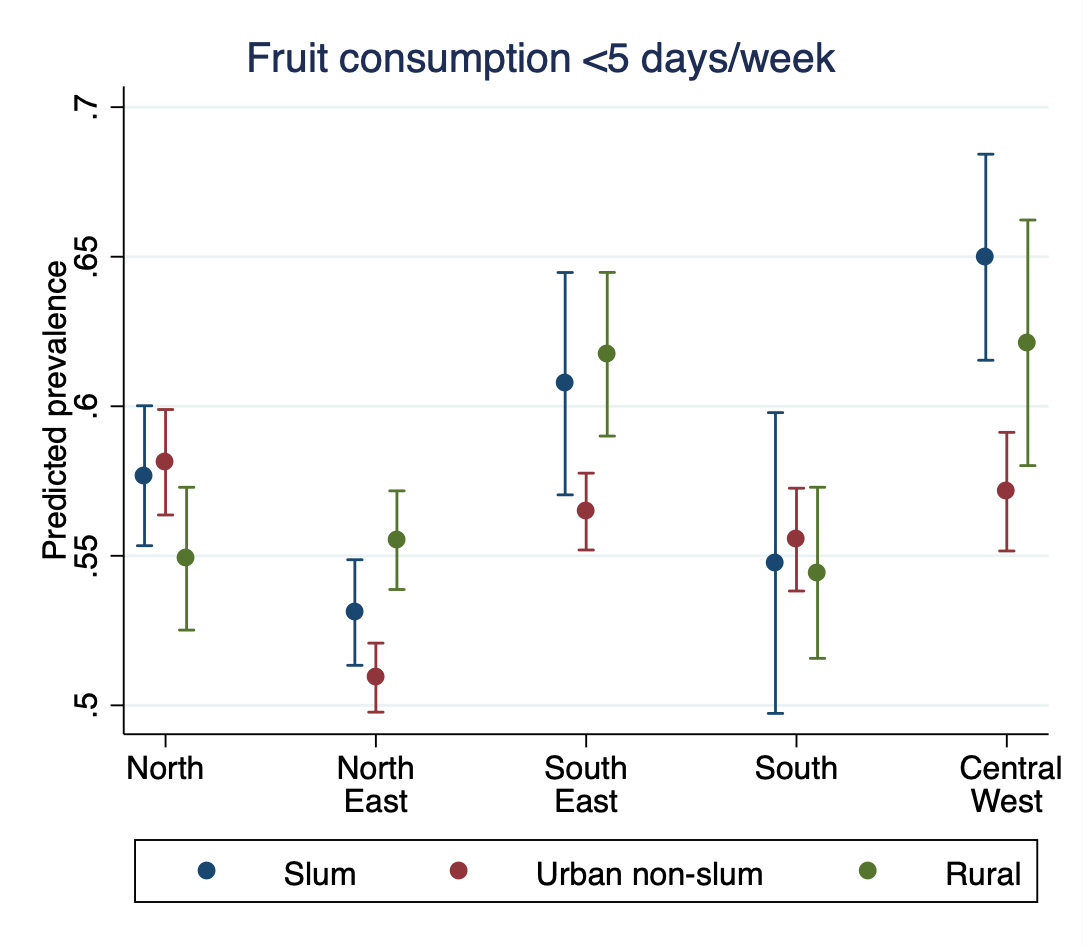 |
| --- | --- |
| 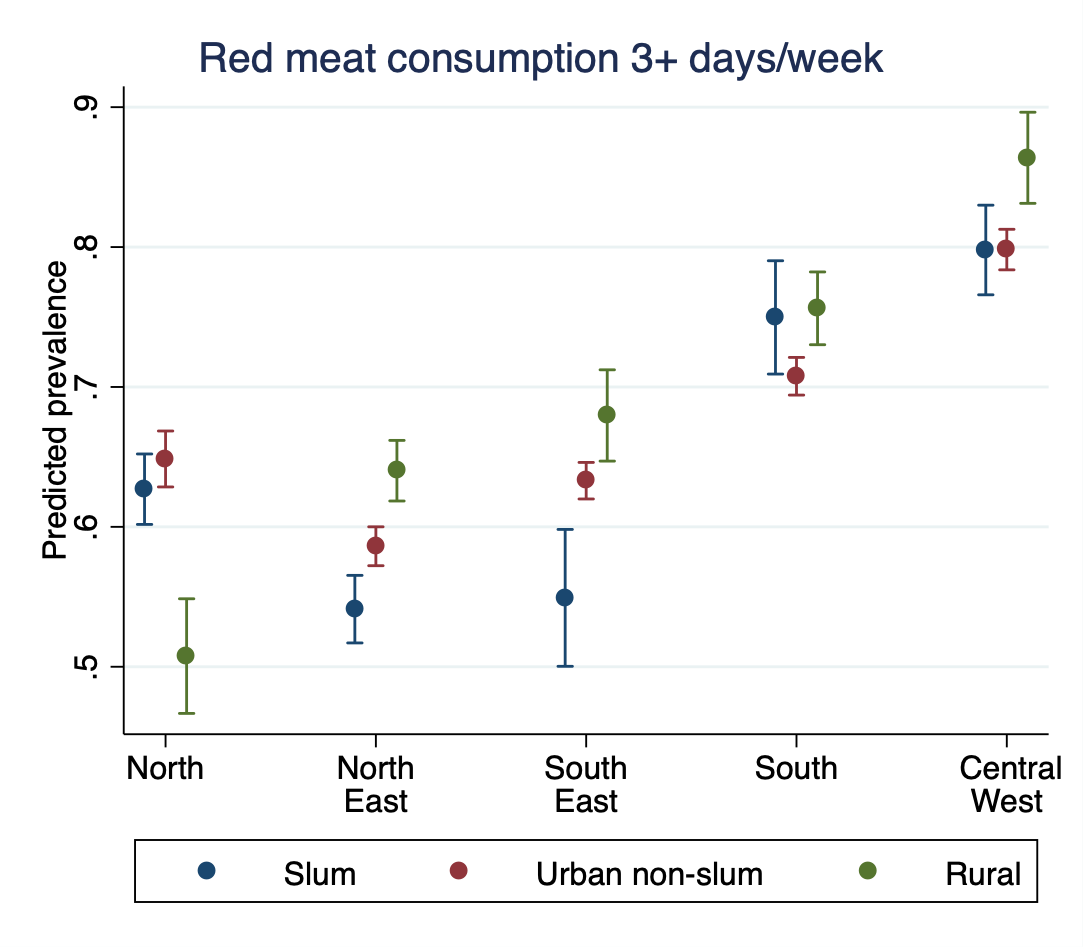 | 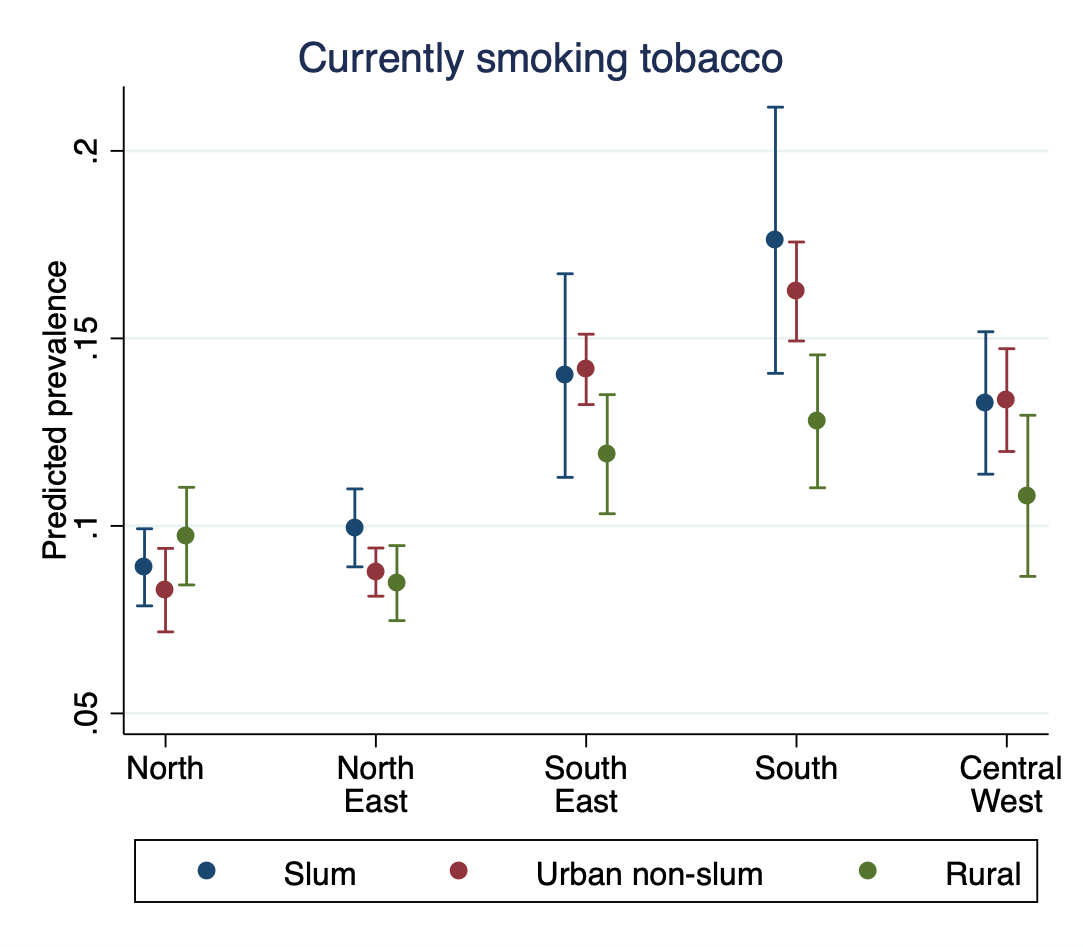 |
| 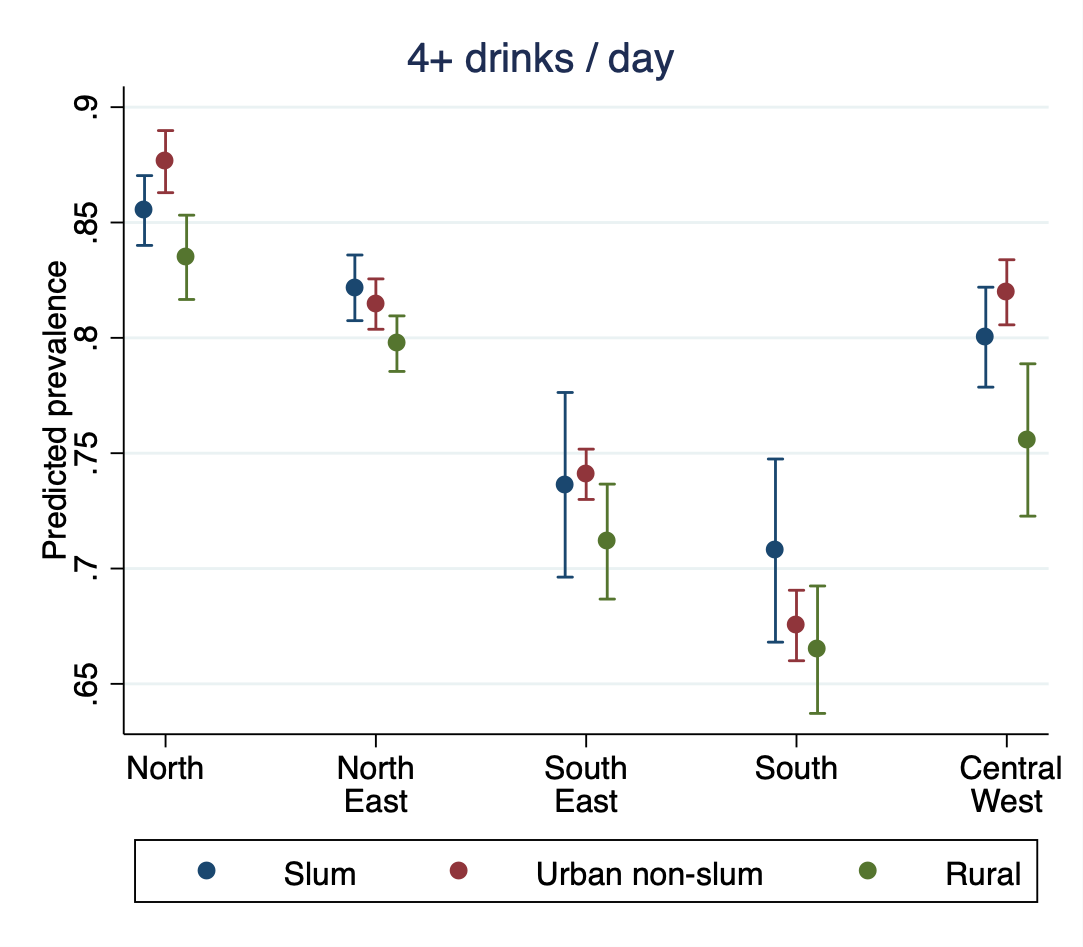 | 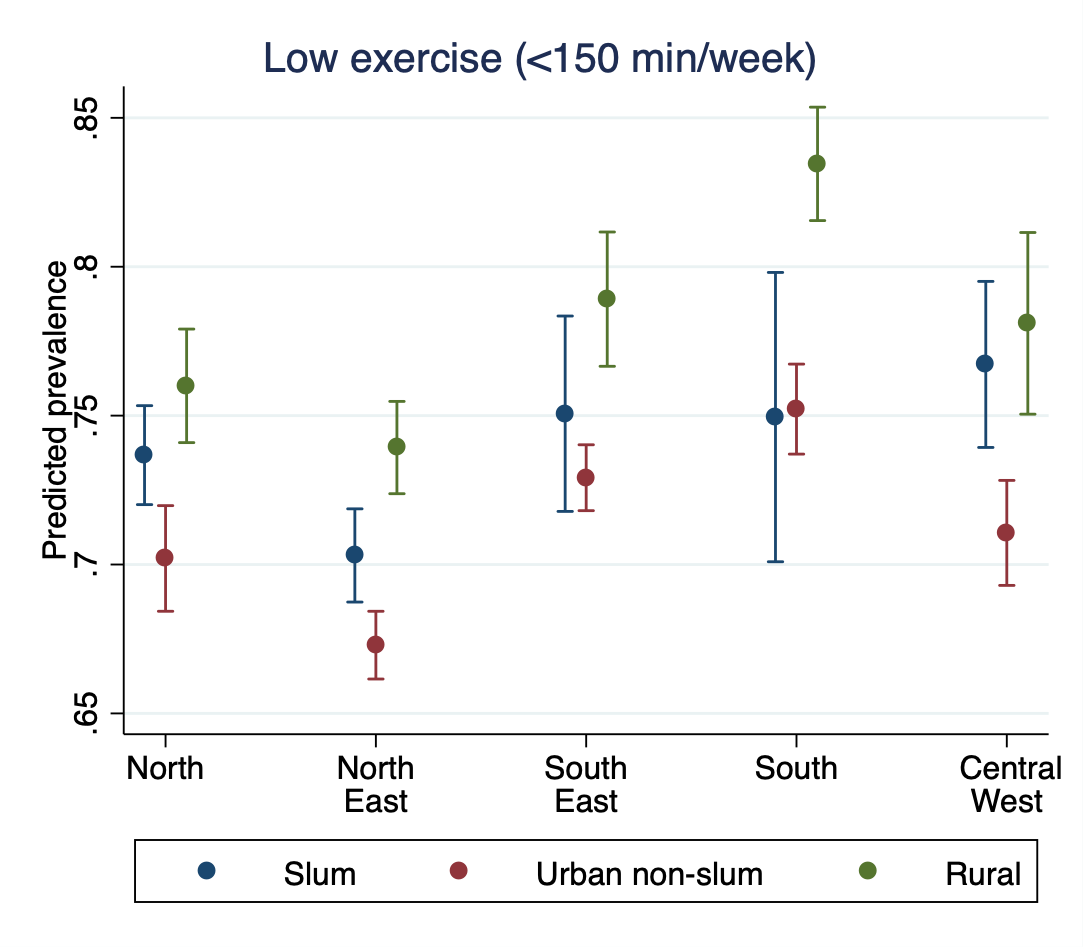 |
| 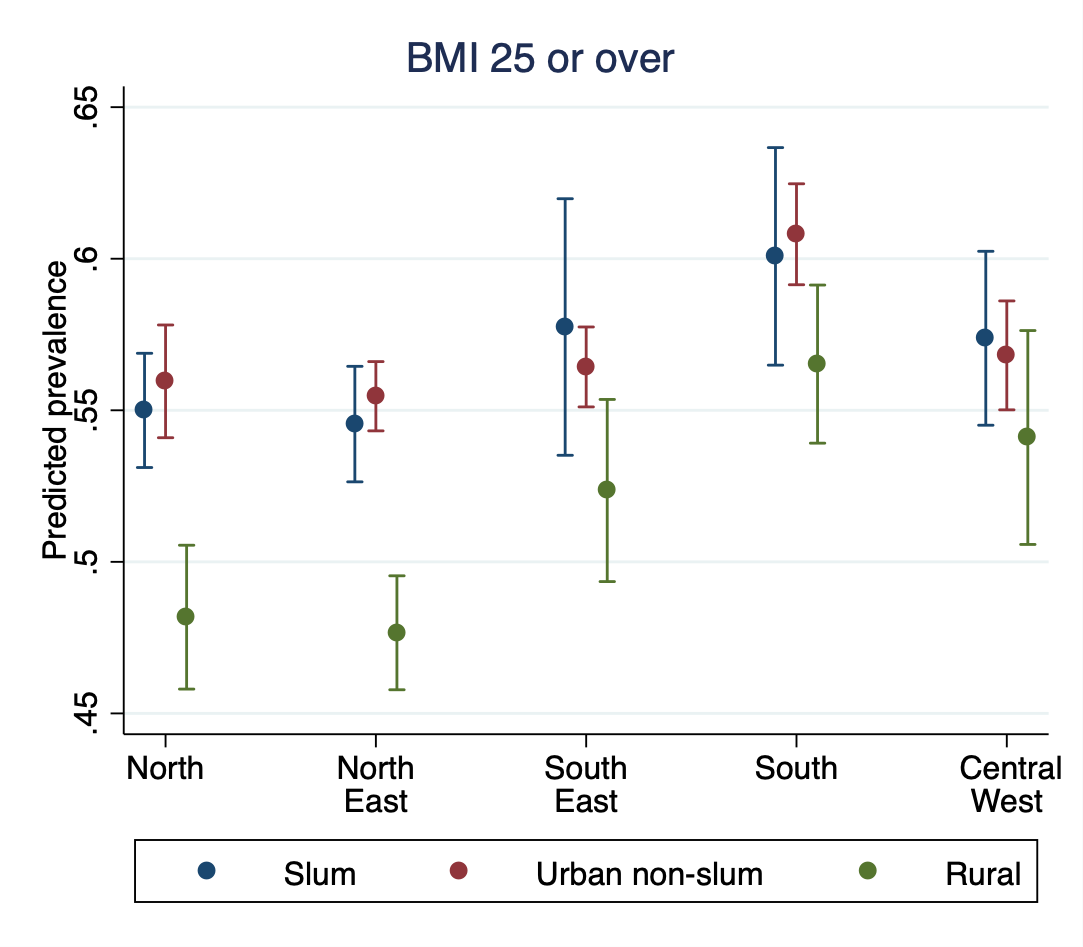 | 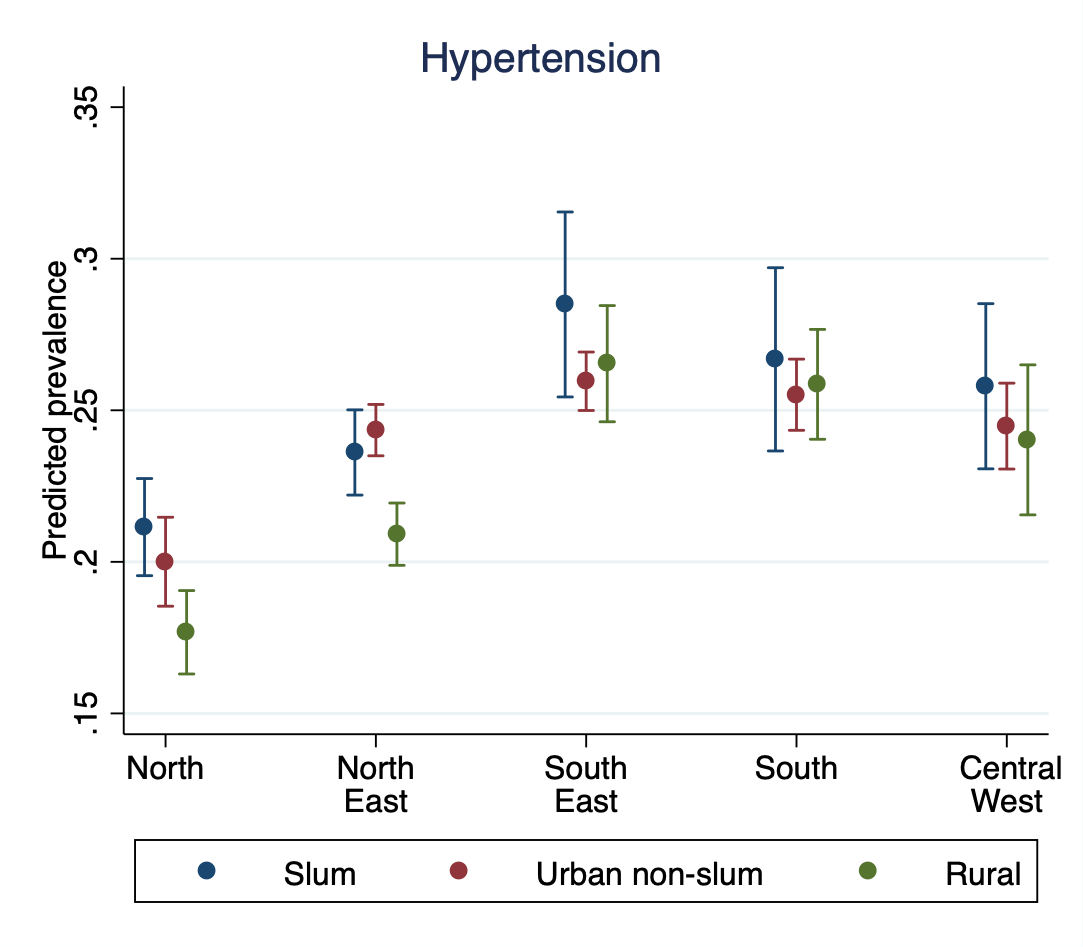 |
| 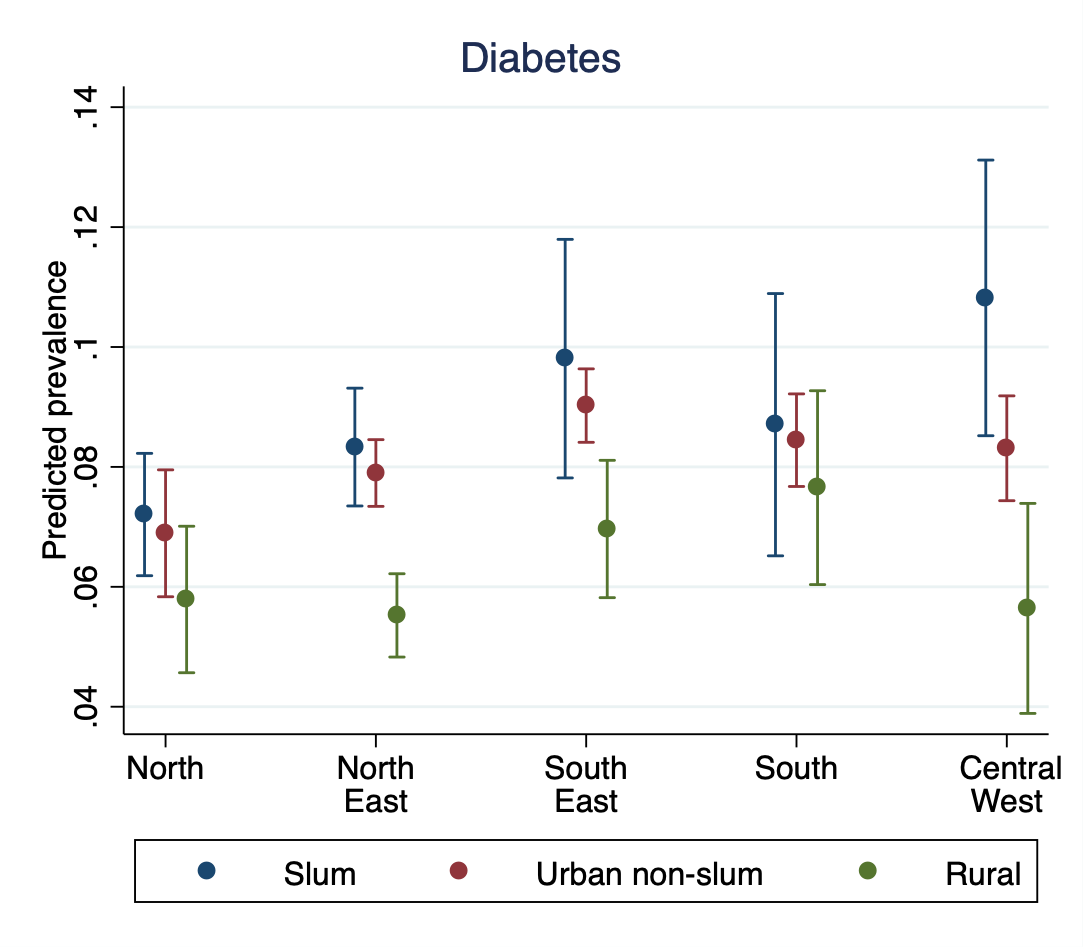 | 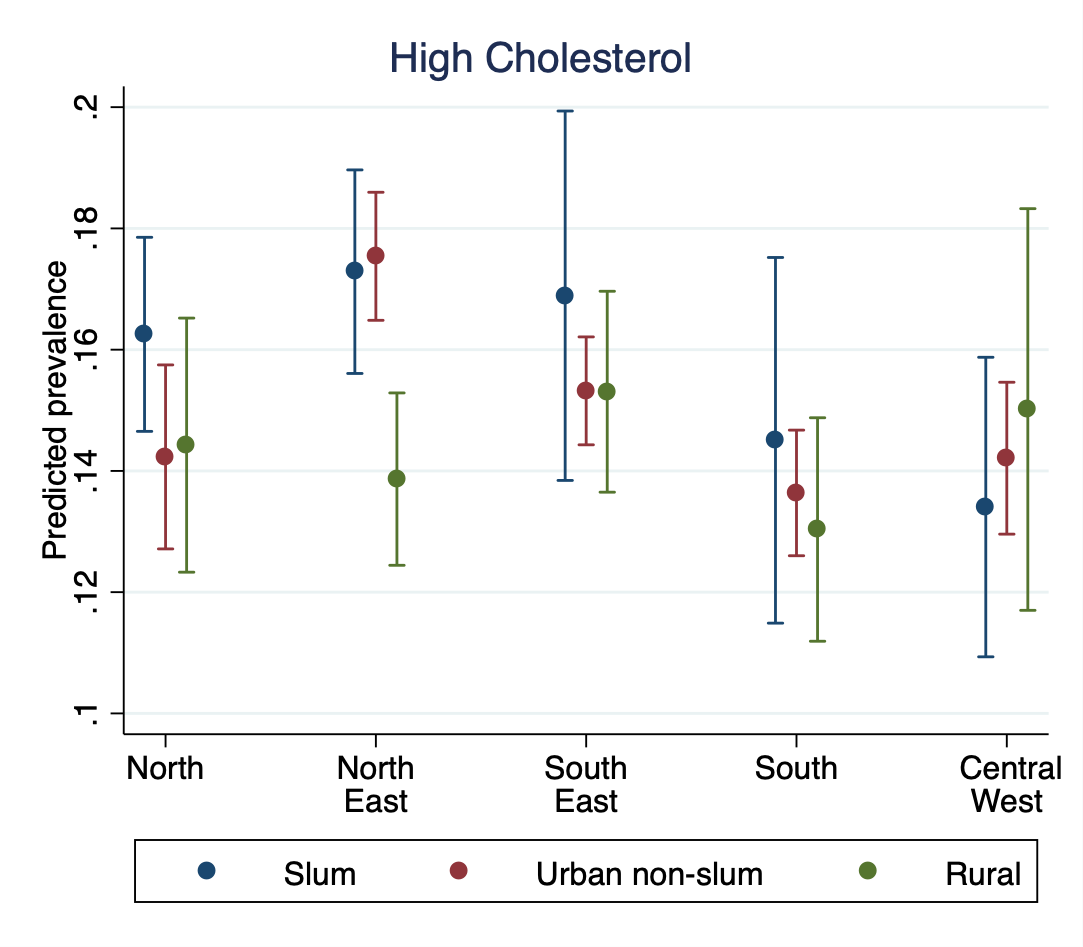 |
| 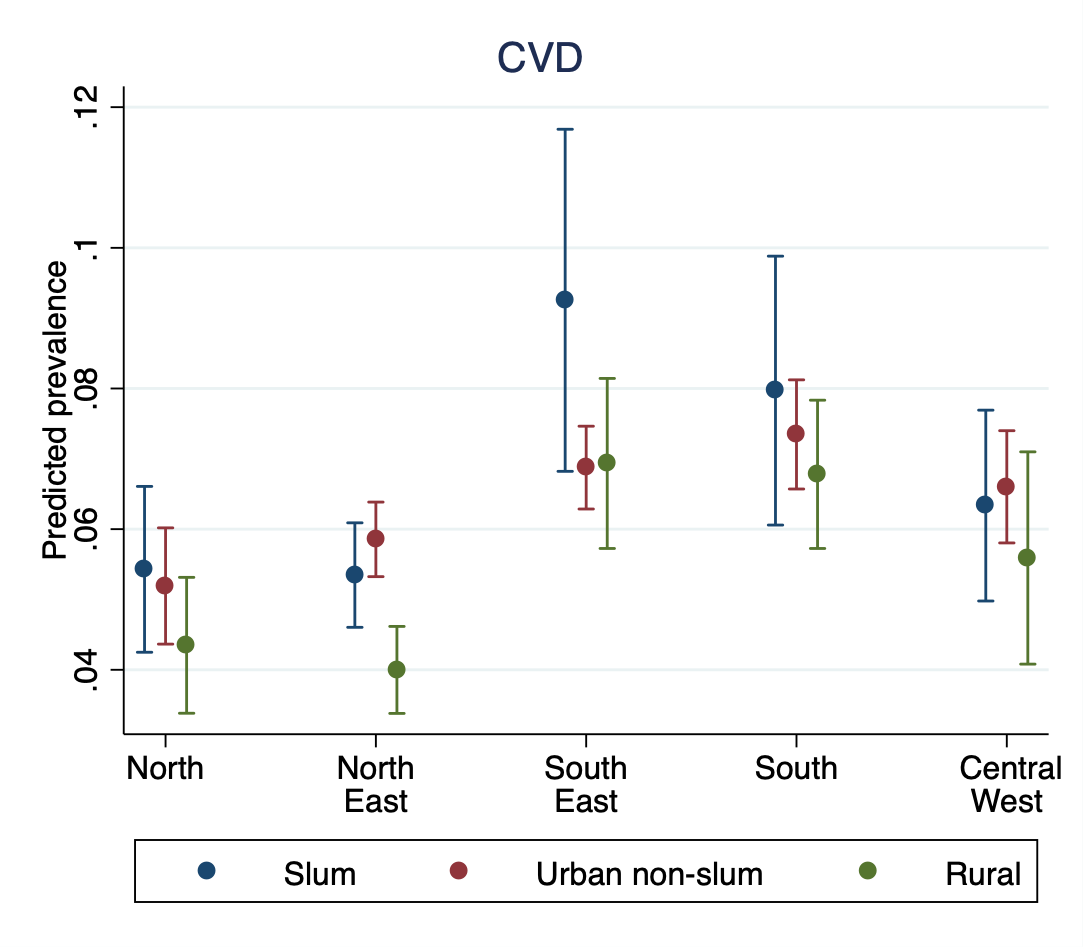 |  |
