# Supplementary material for: Inequalities in the prevalence of cardiovascular disease risk factors in Brazilian slum populations: A cross-sectional study
Source: PLOS Glob Public Health. 2022 Sep 8;2(9):e0000990. doi: 10.1371/journal.pgph.0000990 (PMC10022010; doi:10.1371/journal.pgph.0000990)
Supplement: S1 Table — (DOCX) [file pgph.0000990.s002.docx]

**S1 Table. Unadjusted regression models on the association between slums and behavioural cardiovascular disease risk factors.**

|  | **Low Vegetables Consumption** | **Low Fruits Consumption** | **High Red Meat Consumption** | **Currently Smoke** | **Heavy Alcohol Consumption** | **Low Physical Exercise** |
| --- | --- | --- | --- | --- | --- | --- |
|  | UPR | UPR | UPR | UPR | UPR | UPR |
|  | 95%CI | 95%CI | 95%CI | 95%CI | 95%CI | 95%CI |
| **Area** |  |  |  |  |  |  |
| Non-slum urban (Ref) | 1 | 1 | 1 | 1 | 1 | 1 |
| Slum | 1.256*** | 1.169*** | 0.911*** | 1.079 | 1.115*** | 1.086*** |
|  | 1.214-1.299 | 1.138-1.201 | 0.885-0.937 | 0.997-1.168 | 1.096-1.133 | 1.066-1.106 |
| Rural | 1.359*** | 1.229*** | 0.954*** | 1.089* | 1.089*** | 1.185*** |
|  | 1.317-1.401 | 1.200-1.260 | 0.931-0.977 | 1.009-1.176 | 1.073-1.105 | 1.166-1.204 |
| Number of observations | 90,846 | 90,846 | 90,846 | 90,846 | 90,846 | 90,846 |
|  |  |  |  |  |  |  |
| **Sex** |  |  |  |  |  |  |
| Male (Ref) | 1 | 1 | 1 | 1 | 1 | 1 |
| Female | 0.830*** | 0.826*** | 0.835*** | 0.604*** | 1.065*** | 1.069*** |
|  | 0.809-0.851 | 0.810-0.843 | 0.821-0.849 | 0.568-0.642 | 1.051-1.079 | 1.053-1.086 |
| Number of observations | 90,846 | 90,846 | 90,846 | 90,846 | 90,846 | 90,846 |
|  |  |  |  |  |  |  |
| **Age** |  |  |  |  |  |  |
| 15-29 (Ref) | 1 | 1 | 1 | 1 | 1 | 1 |
| 30-39 | 0.791*** | 0.893*** | 1.002 | 1.238*** | 0.970** | 1.085*** |
|  | 0.761-0.823 | 0.867-0.919 | 0.977-1.027 | 1.114-1.375 | 0.949-0.993 | 1.057-1.115 |
| 40-49 | 0.722*** | 0.808*** | 0.955*** | 1.372*** | 0.959*** | 1.129*** |
|  | 0.694-0.751 | 0.783-0.833 | 0.929-0.981 | 1.229-1.532 | 0.937-0.982 | 1.100-1.160 |
| 50-59 | 0.666*** | 0.730*** | 0.887*** | 1.689*** | 0.957*** | 1.164*** |
|  | 0.639-0.695 | 0.706-0.756 | 0.863-0.913 | 1.520-1.877 | 0.936-0.979 | 1.135-1.194 |
| 60-69 | 0.640*** | 0.637*** | 0.862*** | 1.478*** | 0.989 | 1.195*** |
|  | 0.613-0.668 | 0.613-0.662 | 0.835-0.889 | 1.327-1.646 | 0.967-1.012 | 1.164-1.227 |
| 70+ | 0.599*** | 0.554*** | 0.836*** | 0.810** | 1.064*** | 1.315*** |
|  | 0.570-0.629 | 0.529-0.580 | 0.808-0.865 | 0.708-0.927 | 1.040-1.089 | 1.283-348 |
| Number of observations | 90,846 | 90,846 | 90,846 | 90,846 | 90,846 | 90,846 |
|  |  |  |  |  |  |  |
| **Race/ethnicity** |  |  |  |  |  |  |
| White (Ref) | 1 | 1 | 1 | 1 | 1 | 1 |
| Black | 1.307*** | 1.205*** | 0.933*** | 1.156** | 1.111*** | 1.039** |
|  | 1.253-1.364 | 1.164-0.248 | 0.905-0.962 | 1.044-1.280 | 1.084-1.139 | 1.014-1.064 |
| *Pardo*/mixed (Brown) | 1.300*** | 1.187*** | 0.925*** | 1.142*** | 1.135*** | 1.040*** |
|  | 1.260-1.341 | 1.158-1.216 | 0.907-0.942 | 1.066-1.224 | 1.117-1.153 | 1.022-1.058 |
| Other | 1.097 | 1.063 | 0.848** | 1.050 | 1.095* | 1.043 |
|  | 0.946-1.271 | 0.947-1.193 | 0.760-0.946 | 0.814-1.353 | 1.011-1.187 | 0.980-1.110 |
| Number of observations | 90,846 | 90,846 | 90,846 | 90,846 | 90,846 | 90,846 |
|  |  |  |  |  |  |  |
| **Education level** |  |  |  |  |  |  |
| Illiterate (Ref) | 1 | 1 | 1 | 1 | 1 | 1 |
| Elementary education | 0.844*** | 0.923*** | 1.184*** | 0.928 | 0.901*** | 0.922*** |
|  | 0.810-0.880 | 0.891-0.956 | 1.131-1.239 | 0.843-1.021 | 0.887-0.916 | 0.905-0.940 |
| High school education | 0.842*** | 0.933*** | 1.291*** | 0.567*** | 0.856*** | 0.785*** |
|  | 0.808-0.877 | 0.902-0.966 | 1.235-1.350 | 0.511-0.630 | 0.841-0.872 | 0.768-0.802 |
| Higher education | 0.586*** | 0.678*** | 1.348*** | 0.448*** | 0.712*** | 0.640*** |
|  | 0.555-0.620 | 0.649-0.709 | 1.287-1.412 | 0.396-0.507 | 0.694-0.731 | 0.622-0.659 |
| Number of observations | 90,846 | 90,846 | 90,846 | 90,846 | 90,846 | 90,846 |
|  |  |  |  |  |  |  |
| **Currently employed** |  |  |  |  |  |  |
| Yes (Ref) | 1 | 1 | 1 | 1 | 1 | 1 |
| No | 1.026 | 0.917*** | 0.845*** | 0.797*** | 1.152*** | 1.079*** |
|  | 1.000-1.053 | 0.898-0.937 | 0.829-1.861 | 0.748-0.849 | 1.137-1.167 | 1.062-1.095 |
| Number of observations | 90,846 | 90,846 | 90,846 | 90,846 | 90,846 | 90,846 |
|  |  |  |  |  |  |  |
| **Household income per capita** | |  |  |  |  |  |
| ≤ half minimum wage (Ref) | 1 | 1 | 1 | 1 | 1 | 1 |
| > half but ≤ 1 minimum wage | 0.815*** | 0.856*** | 1.153*** | 0.897** | 0.967*** | 0.957*** |
|  | 0.792-0.839 | 0.836-0.877 | 1.123-1.183 | 0.826-0.973 | 0.952-0.982 | 0.941-0.973 |
| > 1 but ≤ 2 minimum wage | 0.652*** | 0.738*** | 1.243*** | 0.807*** | 0.887*** | 0.903*** |
|  | 0.629-0.675 | 0.718-0.760 | 1.209-1.278 | 0.740-0.878 | 0.871-0.903 | 0.886-0.921 |
| > 2 minimum wage | 0.497*** | 0.570*** | 1.273*** | 0.645*** | 0.755*** | 0.748*** |
|  | 0.474-0.521 | 0.550-0.591 | 1.237-1.310 | 0.586-0.710 | 0.737-0.773 | 0.730-0.767 |
| Number of observations | 90,824 | 90,824 | 90,824 | 90,824 | 90,824 | 90,824 |

Source: Brazilian National Health Survey *Pesquisa Nacional de Saúde* (PNS) 2019. UPR – Unadjusted prevalence ratio; 95%CI – 95% Confidence interval; Ref – Reference category; **p*<0.05; ***p*<0.01; ****p*<0.001; Low Vegetables Consumption – Vegetables consumption <5 days per week; Low Fruits Consumption – Fruits consumption <5 days per week; High Red Meat Consumption – Red meat consumption ≥3 days per week; Currently Smoke – Currently smoke tobacco; Heavy Alcohol Consumption – Alcohol consumption ≥4 per day; Low Physical Exercise – Exercise <150 minutes per week.
