# Supplementary material for: Inequalities in the prevalence of cardiovascular disease risk factors in Brazilian slum populations: A cross-sectional study
Source: PLOS Glob Public Health. 2022 Sep 8;2(9):e0000990. doi: 10.1371/journal.pgph.0000990 (PMC10022010; doi:10.1371/journal.pgph.0000990)
Supplement: S2 Table — (DOCX) [file pgph.0000990.s003.docx]

**S2 Table. Unadjusted regression models on the association between slums and metabolic cardiovascular disease risk factors.**

|  | **Overweight** | **Hypertension** | **Diabetes** | **High Cholesterol** | **CVD** |
| --- | --- | --- | --- | --- | --- |
|  | UPR | UPR | UPR | UPR | UPR |
|  | 95%CI | 95%CI | 95%CI | 95%CI | 95%CI |
| **Area** |  |  |  |  |  |
| Non-slum urban (Ref) | 1 | 1 | 1 | 1 | 1 |
| Slum | 0.966* | 0.978 | 1.051 | 0.972 | 0.968 |
|  | 0.941-0.992 | 0.928-1.031 | 0.956-1.155 | 0.900-1.051 | 0.858-1.091 |
| Rural | 0.857*** | 0.964 | 0.852*** | 0.872*** | 0.852** |
|  | 0.833-0.881 | 0.920-1.011 | 0.775-0.937 | 0.811-0.938 | 0.768-0.945 |
| Number of observations | 89,954 | 88,736 | 84,073 | 82,881 | 90,846 |
|  |  |  |  |  |  |
| **Sex** |  |  |  |  |  |
| Male (Ref) | 1 | 1 | 1 | 1 | 1 |
| Female | 0.973* | 1.376*** | 1.250*** | 1.491*** | 1.105* |
|  | 0.952-0.994 | 1.322-1.432 | 1.154-1.355 | 1.408-1.577 | 1.016-1.201 |
| Number of observations | 89,954 | 88,736 | 84,073 | 82,881 | 90,846 |
|  |  |  |  |  |  |
| **Age** |  |  |  |  |  |
| 15-29 (Ref) | 1 | 1 | 1 | 1 | 1 |
| 30-39 | 1.700*** | 2.923*** | 2.763*** | 1.659*** | 1.506* |
|  | 1.634-1.769 | 2.541-3.363 | 2.030-3.762 | 1.401-1.963 | 1.098-2.065 |
| 40-49 | 1.800*** | 5.746*** | 5.440*** | 3.011*** | 2.646*** |
|  | 1.727-1.876 | 4.985-6.623 | 4.084-7.246 | 2.584-3.509 | 1.986-3.525 |
| 50-59 | 1.764*** | 9.576*** | 12.14*** | 4.700*** | 5.304*** |
|  | 1.688-1.845 | 8.444-10.86 | 9.183-16.04 | 4.060-5.443 | 4.045-6.953 |
| 60-69 | 1.750*** | 13.64*** | 19.52*** | 5.981*** | 8.544*** |
|  | 1.675-1.829 | 12.05-15.45 | 14.90-25.58 | 5.179-6.907 | 6.512-11.21 |
| 70+ | 1.497*** | 16.43*** | 22.69*** | 5.423*** | 13.88*** |
|  | 1.427-1.570 | 14.52-18.58 | 17.24-29.86 | 4.663-6.307 | 10.64-18.11 |
| Number of observations | 89,954 | 88,736 | 84,073 | 82,881 | 90,846 |
|  |  |  |  |  |  |
| **Race/ethnicity** |  |  |  |  |  |
| White (Ref) | 1 | 1 | 1 | 1 | 1 |
| Black | 1.012 | 1.086** | 1.023 | 0.849** | 0.876* |
|  | 0.978-1.048 | 1.021-1.156 | 0.909-1.152 | 0.767-0.939 | 0.769-0.997 |
| *Pardo*/mixed (Brown) | 0.966** | 0.952* | 0.953 | 0.853*** | 0.819*** |
|  | 0.942-0.990 | 0.913-0.993 | 0.877-1.035 | 0.804-0.905 | 0.749-0.896 |
| Other | 0.908 | 1.100 | 1.378 | 0.937 | 0.954 |
|  | 0.808-1.020 | 0.917-1.318 | 0.998-1.902 | 0.710-1.236 | 0.655-1.390 |
| Number of observations | 89,954 | 88,736 | 84,073 | 82,881 | 90,846 |
|  |  |  |  |  |  |
| **Education level** |  |  |  |  |  |
| Illiterate (Ref) | 1 | 1 | 1 | 1 | 1 |
| Elementary education | 1.047* | 0.720*** | 0.682*** | 0.854*** | 0.661*** |
|  | 1.003-1.093 | 0.684-0.757 | 0.613-0.759 | 0.781-0.934 | 0.584-0.748 |
| High school education | 1.001 | 0.364*** | 0.287*** | 0.529*** | 0.318*** |
|  | 0.955-1.048 | 0.341-0.387 | 0.253-0.326 | 0.479-0.584 | 0.273-0.371 |
| Higher education | 1.065** | 0.378*** | 0.271*** | 0.653*** | 0.339*** |
|  | 1.018-1.115 | 0.352-0.406 | 0.235-0.312 | 0.589-0.724 | 0.291-0.396 |
| Number of observations | 89,954 | 88,736 | 84,073 | 82,881 | 90,846 |
|  |  |  |  |  |  |
| **Currently employed** |  |  |  |  |  |
| Yes (Ref) | 1 | 1 | 1 | 1 | 1 |
| No | 0.904*** | 2.088*** | 2.748*** | 1.704*** | 3.011*** |
|  | 0.883-0.924 | 2.008-2.171 | 2.549-2.964 | 1.617-1.795 | 2.768-3.274 |
| Number of observations | 89,954 | 88,736 | 84,073 | 82,881 | 90,846 |
|  |  |  |  |  |  |
| **Household income per capita** | |  |  |  |  |
| ≤ half minimum wage (Ref) | 1 | 1 | 1 | 1 | 1 |
| > half but ≤ 1 minimum wage | 1.104*** | 1.372*** | 1.396*** | 1.326*** | 1.550*** |
|  | 1.073-1.137 | 1.295-1.454 | 1.256-1.552 | 1.213-1.450 | 1.362-1.765 |
| > 1 but ≤ 2 minimum wage | 1.179*** | 1.352*** | 1.333*** | 1.466*** | 1.438*** |
|  | 1.144-1.215 | 1.277-1.431 | 1.198-1.483 | 1.344-1.600 | 1.252-1.651 |
| > 2 minimum wage | 1.179*** | 1.307*** | 1.309*** | 1.643*** | 1.474*** |
|  | 1.143-1.216 | 1.226-1.393 | 1.155-1.483 | 1.496-1.805 | 1.276-1.703 |
| Number of observations | 89,933 | 88,714 | 84,052 | 82,860 | 90,824 |

Source: Brazilian National Health Survey *Pesquisa Nacional de Saúde* (PNS) 2019. UPR – Unadjusted prevalence ratio; 95%CI – 95% Confidence interval; Ref – Reference category; **p*<0.05; ***p*<0.01; ****p*<0.001; CVD – doctor-diagnosed with either heart disease or stroke.
