# Supplementary material for: Inequalities in the prevalence of cardiovascular disease risk factors in Brazilian slum populations: A cross-sectional study
Source: PLOS Glob Public Health. 2022 Sep 8;2(9):e0000990. doi: 10.1371/journal.pgph.0000990 (PMC10022010; doi:10.1371/journal.pgph.0000990)
Supplement: S3 Table — (DOC) [file pgph.0000990.s004.doc]

STROBE Statement—Checklist of items that should be included in reports of ***cross-sectional studies***

|  | Item No | Recommendation |
| --- | --- | --- |
| **Title and abstract** | 1 | (*a*) Indicate the study’s design with a commonly used term in the title or the abstract  Cross-sectional study stated in the title and Methods on page 6 |
|  |  | (*b*) Provide in the abstract an informative and balanced summary of what was done and what was found  Provided in Abstract on page 2 and 3 |
| Introduction | | |
| Background/rationale | 2 | Explain the scientific background and rationale for the investigation being reported  Included in Introduction on page 4, 5 and 6 |
| Objectives | 3 | State specific objectives, including any prespecified hypotheses  Included in Introduction on page 6 |
| Methods | | |
| Study design | 4 | Present key elements of study design early in the paper  Included in Methods on page 6 and 7 |
| Setting | 5 | Describe the setting, locations, and relevant dates, including periods of recruitment, exposure, follow-up, and data collection  Included in Methods on page 7 |
| Participants | 6 | (*a*) Give the eligibility criteria, and the sources and methods of selection of participants  Included in Methods on page 7 |
| Variables | 7 | Clearly define all outcomes, exposures, predictors, potential confounders, and effect modifiers. Give diagnostic criteria, if applicable  Included in Methods on page 7, 8, 9 and 10 |
| Data sources/ measurement | 8* | For each variable of interest, give sources of data and details of methods of assessment (measurement). Describe comparability of assessment methods if there is more than one group  Included in Methods on page 7, 8, 9 and 10 |
| Bias | 9 | Describe any efforts to address potential sources of bias  Addressed in Methods on page 7 |
| Study size | 10 | Explain how the study size was arrived at  Included in Methods on page 7 |
| Quantitative variables | 11 | Explain how quantitative variables were handled in the analyses. If applicable, describe which groupings were chosen and why  Included in Methods on page 7, 8, 9 and 10 |
| Statistical methods | 12 | (*a*) Describe all statistical methods, including those used to control for confounding  Included in Methods on page 10 and 11 |
|  |  | (*b*) Describe any methods used to examine subgroups and interactions  Included in Methods on page 10 and 11 |
|  |  | (*c*) Explain how missing data were addressed  Included in Methods on page 7 |
|  |  | (*d*) If applicable, describe analytical methods taking account of sampling strategy  Included in Methods on page 7 |
|  |  | (*e*) Describe any sensitivity analyses  Included in Methods on page 10 and 11 |
| Results | | |
| Participants | 13* | (a) Report numbers of individuals at each stage of study—eg numbers potentially eligible, examined for eligibility, confirmed eligible, included in the study, completing follow-up, and analysed  Included in Results on Table 2 |
|  |  | (b) Give reasons for non-participation at each stage  Addressed in study limitations paragraph on page 21 and 22 |
|  |  | (c) Consider use of a flow diagram  Not applicable |
| Descriptive data | 14* | (a) Give characteristics of study participants (eg demographic, clinical, social) and information on exposures and potential confounders  Included in Results on Table 3, Figure 1 and 2; and page 11, 12, 13 and 14 |
|  |  | (b) Indicate number of participants with missing data for each variable of interest  Not applicable |
| Outcome data | 15* | Report numbers of outcome events or summary measures  Included in Results on Table 3, Figure 1 and 2; and page 11, 12, 13 and 14 |
| Main results | 16 | (*a*) Give unadjusted estimates and, if applicable, confounder-adjusted estimates and their precision (eg, 95% confidence interval). Make clear which confounders were adjusted for and why they were included  Included in Results on Table 4 and 5; and page 14, 16 and 17 |
|  |  | (*b*) Report category boundaries when continuous variables were categorized  Included in Results on Table 4 and 5 |
|  |  | (*c*) If relevant, consider translating estimates of relative risk into absolute risk for a meaningful time period  Used adjusted prevalence ratio (APR) |
| Other analyses | 17 | Report other analyses done—eg analyses of subgroups and interactions, and sensitivity analyses  Included in S1 and S2 Table, and S1 Figure |
| Discussion | | |
| Key results | 18 | Summarise key results with reference to study objectives  Included in Discussion on page 19 |
| Limitations | 19 | Discuss limitations of the study, taking into account sources of potential bias or imprecision. Discuss both direction and magnitude of any potential bias  Included in Discussion on page 21 and 22 |
| Interpretation | 20 | Give a cautious overall interpretation of results considering objectives, limitations, multiplicity of analyses, results from similar studies, and other relevant evidence  Included in Discussion on page 20 and 21 |
| Generalisability | 21 | Discuss the generalisability (external validity) of the study results  Included in Discussion on page 22 and 23 |
| Other information | | |
| Funding | 22 | Give the source of funding and the role of the funders for the present study and, if applicable, for the original study on which the present article is based  Not applicable – the authors did not receive any funding |

*Give information separately for exposed and unexposed groups.

**Note:** An Explanation and Elaboration article discusses each checklist item and gives methodological background and published examples of transparent reporting. The STROBE checklist is best used in conjunction with this article (freely available on the Web sites of PLoS Medicine at http://www.plosmedicine.org/, Annals of Internal Medicine at http://www.annals.org/, and Epidemiology at http://www.epidem.com/). Information on the STROBE Initiative is available at www.strobe-statement.org.
