# Supplementary material for: Inequalities in the prevalence of cardiovascular disease risk factors in Brazilian slum populations: A cross-sectional study
Source: PLOS Glob Public Health. 2022 Sep 8;2(9):e0000990. doi: 10.1371/journal.pgph.0000990 (PMC10022010; doi:10.1371/journal.pgph.0000990)
Supplement: Attachment — Submitted filename: Response to Reviewers.docx [file pgph.0000990.s005.docx]

**Jasper Chan**

School of Public Health – Imperial College London

Medical School Building, St Mary’s Campus

Norfolk Place, London, W2 1PG

United Kingdom

jasper.chan17@imperial.ac.uk

+44 7795 895 916

**Dear Reviewers,**

We thank the reviewers for their helpful and valuable inputs to the manuscript. Below are our responses to the points raised by the reviewers. We hope that we have satisfyingly addressed them and that the manuscript will now be suited for publication.

Yours faithfully,

**Jasper Chan,**

**on behalf of the authors.**

The changes have been made for the addition of an author summary (page 3) and the ethics statement (page 6).

**Reviewer #1: In this manuscript Chan et al., present the results of their analysis of inequalities in the prevalence of self-reported cardiovascular disease risk factors (CVRFs, both behavioral and doctor-diagnosed) attributable to demographic and socioeconomic factors, specifically examining the inequalities between slum and non-slum inhabitants, using for this purpose publicly available data from the 2019 Brazilian National Health Survey (BNHS). This secondary analysis is innovative in documenting the burden, distribution, and clustering of CVRFs among marginalized populations studied in a well-designed and conducted nationwide representative survey, whose results have important implications for public health policy in the Latin American region, considering the large fraction of population currently living in the precarious conditions that characterize slums.

The main exposure of the analysis is residence as compared to non-residence in a slum, both levels corresponding to urban settings; however, the data set also provides information about rural dwellers which was incorporated into the analysis and reported in the results’ section but without further discussion in the corresponding section. If there was no hypothesis behind rural residency as an exposure, what was the reason for keeping it in the analysis? I am not suggesting that rural residency is not an interesting and important exposure, in fact, rural-urban inequities in CVFRs are rampant in Latin America and your results support this point; however, considering the focus of your analysis, I suggest either developing the discussion about the findings on the rural-urban contrast or excluding rural residency from the exposure at all. The latest can be done by creating a subpopulation within the data set that contains the levels "slum" and "non-slum" (both urban) and conduct the analysis in that subset without excluding rural dwellers.**

We appreciate these comments and have introduced edits as suggested by the reviewer. We have expanded discussion of the rural-urban contrast and have highlighted the rural results more in the results and discussion.

**From the statistical point of view, in estimating prevalence the authors correctly accounted for the sampling design of the survey by incorporating sampling weights (and presumably, clustering); however, at the time of estimating associations, it is not clear to me why they opted for the odds ratios (from logistic regression) instead of prevalence ratios (from, for instance, binomial regression) considering that the first measure of association overestimates the magnitude of relationships in scenarios where outcomes are highly prevalent, as is the case for most of the CVRFs. Also, prevalence ratios are more “natural” estimates of what it is being studied from a cross-sectional design and easier to communicate than odds ratios to the attempted audience of the paper. In consequence, I strongly recommend estimating and presenting prevalence ratios instead of odds ratios.**

Thank you for this suggestion. We agree with the reviewer that prevalence ratios are more appropriate. We have now repeated our analyses using Poisson regression models to obtain PRs. The methods and results have all been updated – there have been no significant changes to our main findings.

**The discussion, in what refers to the "slum" versus "non-slum" residency contrast, is focused on the main findings of the analysis and framed in the most relevant evidence, pondering the results in light of the limitations of a cross-sectional design and self-reported data on exposures and health outcomes; however, I do not consider the use of regression models a limitation but a strength of the analysis (see lines 388-391). Finally, the authors did an excellent job at developing both policy and clinical implications for their findings.**

Thank you for these positive comments. We have removed the text relating to regression modelling as a limitation.

**Minor comments.

In the “Data analysis” section consider making explicit how the complex sampling design of the survey was incorporated into the analysis (i.e., clustering), as well as which variables were included into the adjusted models (only stated in line 389).**

We have introduced text in the data analysis section:

“All analyses accounted for clustered sampling of the survey and weighting for non-response.”

“…all models were adjusted for location of residence (urban slum, urban non-slum, rural), sex, age, race/ethnicity, educational level, employment status, and household income per capita.”

**In the “Results” section, line 230, perhaps the authors meant to say, “Over two thirds…”.**

This change has been made.

**Reviewer #2: This manuscript assessed data from Brazil's most recent health demographic survey to appraise the association between cardiovascular risk factors and housing conditions. The theme is relevant to global public health; the study is well organized and reads smoothly. This reviewer agrees with the main methodological options adopted here, with special mention to the choice of the database and the statistical analysis. Also, the robust assertion about study limitations is a strong point.
I also underscore the original use of the survey’s data to classify slum households according to UN-Habitat criteria as a study’s strength, which can instruct further research on the subject.
Aiming to contribute to the authors and improve even further this excellent manuscript, I offer two discretionary suggestions.
1. I missed the report on unadjusted odds ratios as supplemental tables. I understand the difficulty of reporting too many regression models. The study collates eleven outcome variables and seven factors. However, socioeconomic factors such as education, employment status, and income correlate among themselves. The problem increases when we consider that race/ethnicity and slum residence can also be associated with the remaining socioeconomic covariates. Excessive autocorrelation can interfere with regression models’ goodness-of-fit indicators. Assessing variance inflation, direct acyclic graphs, and comparing unadjusted and adjusted regression results are tools to attenuate this problem.**

These are valuable suggestions. As suggested, we now include text relating to VIFs in the methods and have included unadjusted estimates in the supplementary material.

“Collinearity of variables were tested and found to be unproblematic as all variance inflation factors were less than 2.”

**2. Brazil is a large country with substantial geographic inequalities. I wonder whether the results reported here would hold for the poorer North and Northeast regions to the same extent as the remaining, wealthier regions. The database is sufficiently large and allows this assessment. Maybe the authors will not have difficulties running the same analytical routines for each geographic region and reporting the results in supplemental tables if the results are eventually relevant.**

This is a useful suggestion and we have carried out a supplementary analysis exploring interactions with region of residence. We have updated the manuscript exploring these findings.
