# Supplementary material for: Inequalities in the prevalence of cardiovascular disease risk factors in Brazilian slum populations: A cross-sectional study
Source: PLOS Glob Public Health. 2022 Sep 8;2(9):e0000990. doi: 10.1371/journal.pgph.0000990 (PMC10022010; doi:10.1371/journal.pgph.0000990)
Supplement: Attachment — Submitted filename: Response to Reviewers II.docx [file pgph.0000990.s006.docx]

We thank the reviewers for their helpful and valuable inputs to the manuscript. Below are our responses to the points raised by the reviewers.

**Reviewer #3: This is an interesting and well-written manuscript about the relationship between residence in a slum and two forms of CVD risk factors (behavioral and doctor-diagnosed conditions) from the 2019 PNS. While the paper is clear and thorough, there are a few ways it may be further strengthened:
1. Has this approach to defining a slum resident been validated in prior studies (not the definition persay, but this retrospective attempt to define urban residents as slum-dwellers). in the limitations, the authors alluded to prior studies that did this but would be even more convincing if they said how well this approach performed previously.**

Thank you for the positive comments and raising this important point. To our knowledge there are no validation studies of this approach to defining slum inhabitants, although this approach has been used in multiple studies and by UN organisations for assessing slum populations and their health and socioeconomic outcomes. Unfortunately, it is not possible to indicate how well these approaches perform, but we have now acknowledged this in the limitations:

“While no validation studies of this approach for identifying slum inhabitants have been carried out, we used the UN-Habitat definition, which is widely accepted and has been used in previously published work for assessing slum populations and their health [13,49,50,75,76].” (page 22)

UN-Habitat definition of slums can be found here:

- United Nations Human Settlement Programme (UN-Habitat). *SDG Indicator 11.1.1 Training Module: Adequate Housing and Slum Upgrading.* UN-Habitat. 2018.

These are some of the studies which have used the UN-Habitat definition of slums:

- Ezeh A, Oyebode O, Satterthwaite D, Chen Y, Ndugwa R, Sartori J, et al. The history, geography, and sociology of slums and the health problems of people who live in slums. *Lancet.* 2017; 389 (10068): 547-558. Available from: doi: 10.1016/S0140-6736(16)31650-6.
- Pitcairn CFM, Laverty AA, Chan JJL, Oyebode O, Mrejen M, Pescarini J, Machado DB, Hone TV. Inequalities in the prevalence of major depressive disorder in Brazilian slum populations: a cross-sectional analysis. *Epidemiology and Psychiatric Sciences.* 2021; 30: e66. Available from: doi: 10.1017/S204579602100055X.
- Hacker KP, Seto KC, Costa F, Corburn J, Reis MG, Ko AI, Diuk-Wasser MA. Urban slum structure: integrating socioeconomic and land cover data to model slum evolution in Salvador, Brazil. *International Journal of Health Geographics.* 2013; 12 (1): 45. Available from: doi: 10.1186/1476-072X-12-45.
- Kuffer M, Thomson DR, Boo G, Mahabir R, Grippa T, Vanhuysse S, Engstrom R, Ndugwa R, Makau J, Darin E, de Albuquerque JP, Kabaria. The Role of Earth Observation in an Integrated Deprived Area Mapping “System” for Low-to-Middle-Income Countries. *Remote Sensing.* 2020; 12 (6): 982. Available from: doi: 10.3390/rs12060982.
- Brotherhood L, Cavalcanti T, Da Mata D, Santos C. Slums and pandemics. *Journal of Development Economics*. 2022; 157 (102882). Available from: doi: 10.1016/j.jdeveco.2022.102882.

**2. Additionally, the derivation of the four of the metabolic risk factors was a little bit unclear. Was BMI measured in the survey and used to define overweight? Were the "doctor diagnosed" conditions only self-report (the limitations indicates this is likely the case)? Though mentioned in the limitations, this seems pretty important. Were there biomarkers measured in the survey that could further bolster these definitions or be used as a second way to categorize people as having these risk factors?**

We have amended the methods to improve clarity. Unfortunately, biomarker data was not available.

“Both height and weight were measured at the interview to determine BMI. The doctor-diagnosed conditions were determined in the interview as: “Has a doctor ever diagnosed you with…” (page 8)

**3. Table 3 might benefit from the addition of a statistical comparison test.**

We do not feel a statistical test is necessary here as we are not explicitly testing the differences in socioeconomic differences between rural, urban non-slum, and urban slum populations. It is unlikely to provide much value to readers. Any differences in socioeconomic characteristics between populations that could explain differences in the outcomes are captured during regression analyses, and we focus on these as our main results.

**4. The inclusion of the rural population as a comparison is interesting but sometimes may distract from the focus on slum residents. I wonder if the authors should have made the findings for rural residents (as a comparison) more explicit in the paper and especially in the discussion.**

We thank the reviewer for this comment, but we have already responded to this comment for a previous reviewer. We have already expanded the discussion on the rural-urban contrast and have highlighted the rural results more in the results and discussion. We believe further alterations to the text will cloud the main messages, and make interpretation more difficult.
