# Supplementary material for: Inequalities in the prevalence of cardiovascular disease risk factors in Brazilian slum populations: A cross-sectional study
Source: PLOS Glob Public Health. 2022 Sep 8;2(9):e0000990. doi: 10.1371/journal.pgph.0000990 (PMC10022010; doi:10.1371/journal.pgph.0000990)
Supplement: Attachment — Submitted filename: Response to Reviewers III.docx [file pgph.0000990.s007.docx]

**Dear Editor,**

We thank the editor for his recommendation. We have now added the STROBE checklist for cross-sectional studies to our supplementary files.

We hope the manuscript will now be suited for publication.

Yours faithfully,

**Jasper Chan,**

**on behalf of the authors.**
